# Structural inequities contribute to racial/ethnic differences in neurophysiological tone, but not threat reactivity, after trauma exposure

**DOI:** 10.1038/s41380-023-01971-x

**Published:** 2023-02-01

**Authors:** Nathaniel G. Harnett, Negar Fani, Sierra Carter, Leon D. Sanchez, Grace E. Rowland, William M. Davie, Camilo Guzman, Lauren A. M. Lebois, Timothy D. Ely, Sanne J. H. van Rooij, Antonia V. Seligowski, Sterling Winters, Lana R. Grasser, Paul I. Musey, Mark J. Seamon, Stacey L. House, Francesca L. Beaudoin, Xinming An, Donglin Zeng, Thomas C. Neylan, Gari D. Clifford, Sarah D. Linnstaedt, Laura T. Germine, Kenneth A. Bollen, Scott L. Rauch, John P. Haran, Alan B. Storrow, Christopher Lewandowski, Phyllis L. Hendry, Sophia Sheikh, Christopher W. Jones, Brittany E. Punches, Robert A. Swor, Lauren A. Hudak, Jose L. Pascual, Erica Harris, Anna M. Chang, Claire Pearson, David A. Peak, Roland C. Merchant, Robert M. Domeier, Niels K. Rathlev, Steven E. Bruce, Mark W. Miller, Robert H. Pietrzak, Jutta Joormann, Deanna M. Barch, Diego A. Pizzagalli, Steven E. Harte, James M. Elliott, Ronald C. Kessler, Karestan C. Koenen, Samuel A. McLean, Tanja Jovanovic, Jennifer S. Stevens, Kerry J. Ressler

**Affiliations:** 1 Division of Depression and Anxiety, McLean Hospital, Belmont, MA USA; 2 Department of Psychiatry, Harvard Medical School, Boston, MA USA; 3 Department of Psychiatry and Behavioral Sciences, Emory University School of Medicine, Atlanta, GA USA; 4 Department of Psychology, Georgia State University, Atlanta, GA USA; 5 Department of Emergency Medicine, Brigham and Women’s Hospital, Boston, MA USA; 6 Department of Emergency Medicine, Harvard Medical School, Boston, MA USA; 7 Department of Psychiatry and Behavioral Neurosciences, Wayne State University, Detroit, MI USA; 8 Department of Psychiatry, Henry Ford Health System, Detroit, MI USA; 9 Department of Emergency Medicine, Indiana University School of Medicine, Indianapolis, IN USA; 10 Department of Surgery, Division of Traumatology, Surgical Critical Care and Emergency Surgery, University of Pennsylvania, Philadelphia, PA USA; 11 Perelman School of Medicine, University of Pennsylvania, Philadelphia, PA USA; 12 Department of Emergency Medicine, Washington University School of Medicine, St. Louis, MO USA; 13 Department of Epidemiology, Brown University School of Public Health, Providence, RI USA; 14 Institute for Trauma Recovery, Department of Anesthesiology, University of North Carolina at Chapel Hill, Chapel Hill, NC USA; 15 Department of Biostatistics, Gillings School of Global Public Health, University of North Carolina, Chapel Hill, NC USA; 16 Departments of Psychiatry and Neurology, University of California San Francisco, San Francisco, CA USA; 17 Department of Biomedical Informatics, Emory University School of Medicine, Atlanta, GA USA; 18 Department of Biomedical Engineering, Georgia Institute of Technology and Emory University, Atlanta, GA USA; 19 Institute for Technology in Psychiatry, McLean Hospital, Belmont, MA USA; 20 The Many Brains Project, Belmont, MA USA; 21 Department of Psychology and Neuroscience & Department of Sociology, University of North Carolina at Chapel Hill, Chapel Hill, NC USA; 22 Department of Psychiatry, McLean Hospital, Belmont, MA USA; 23 Department of Emergency Medicine, University of Massachusetts Chan Medical School, Worcester, MA USA; 24 Department of Emergency Medicine, Vanderbilt University Medical Center, Nashville, TN USA; 25 Department of Emergency Medicine, Henry Ford Health System, Detroit, MI USA; 26 Department of Emergency Medicine, University of Florida College of Medicine -Jacksonville, Jacksonville, FL USA; 27 Department of Emergency Medicine, Cooper Medical School of Rowan University, Camden, NJ USA; 28 Department of Emergency Medicine, Ohio State University College of Medicine, Columbus, OH USA; 29 Ohio State University College of Nursing, Columbus, OH USA; 30 Department of Emergency Medicine, Oakland University William Beaumont School of Medicine, Rochester, MI USA; 31 Department of Emergency Medicine, Emory University School of Medicine, Atlanta, GA USA; 32 Department of Surgery, Department of Neurosurgery, University of Pennsylvania, Philadelphia, PA USA; 33 Einstein Medical Center, Philadelphia, PA USA; 34 Department of Emergency Medicine, Jefferson University Hospitals, Philadelphia, PA USA; 35 Department of Emergency Medicine, Wayne State University, Ascension St. John Hospital, Detroit, MI USA; 36 Department of Emergency Medicine, Massachusetts General Hospital, Boston, MA USA; 37 Department of Emergency Medicine, Saint Joseph Mercy Hospital, Ypsilanti, MI USA; 38 Department of Emergency Medicine, University of Massachusetts Medical School-Baystate, Springfield, MA USA; 39 Department of Psychological Sciences, University of Missouri - St. Louis, St. Louis, MO USA; 40 National Center for PTSD, Behavioral Science Division, VA Boston Healthcare System, Boston, MA USA; 41 Department of Psychiatry, Boston University School of Medicine, Boston, MA USA; 42 National Center for PTSD, Clinical Neurosciences Division, VA Connecticut Healthcare System, West Haven, CT USA; 43 Department of Psychiatry, Yale School of Medicine, New Haven, CT USA; 44 Department of Psychology, Yale University, New Haven, CT USA; 45 Department of Psychological & Brain Sciences, Washington University in St. Louis, St. Louis, MO USA; 46 Department of Anesthesiology, University of Michigan Medical School, Ann Arbor, MI USA; 47 Department of Internal Medicine-Rheumatology, University of Michigan Medical School, Ann Arbor, MI USA; 48 Kolling Institute, University of Sydney, St Leonards, New South Wales Australia; 49 Faculty of Medicine and Health, University of Sydney, Northern Sydney Local Health District, St Leonards, New South Wales Australia; 50 Physical Therapy & Human Movement Sciences, Feinberg School of Medicine, Northwestern University, Chicago, IL USA; 51 Department of Health Care Policy, Harvard Medical School, Boston, MA USA; 52 Department of Epidemiology, Harvard T.H. Chan School of Public Health, Harvard University, Boston, MA USA; 53 Department of Emergency Medicine, University of North Carolina at Chapel Hill, Chapel Hill, NC USA; 54 Institute for Trauma Recovery, Department of Psychiatry, University of North Carolina at Chapel Hill, Chapel Hill, NC USA

**Keywords:** Psychiatric disorders, Psychology, Neuroscience, Prognostic markers

## Abstract

Considerable racial/ethnic disparities persist in exposure to life stressors and socioeconomic resources that can directly affect threat neurocircuitry, particularly the amygdala, that partially mediates susceptibility to adverse posttraumatic outcomes. Limited work to date, however, has investigated potential racial/ethnic variability in amygdala reactivity or connectivity that may in turn be related to outcomes such as post-traumatic stress disorder (PTSD). Participants from the AURORA study (*n* = 283), a multisite longitudinal study of trauma outcomes, completed functional magnetic resonance imaging and psychophysiology within approximately two-weeks of trauma exposure. Seed-based amygdala connectivity and amygdala reactivity during passive viewing of fearful and neutral faces were assessed during fMRI. Physiological activity was assessed during Pavlovian threat conditioning. Participants also reported the severity of posttraumatic symptoms 3 and 6 months after trauma. Black individuals showed lower baseline skin conductance levels and startle compared to White individuals, but no differences were observed in physiological reactions to threat. Further, Hispanic and Black participants showed greater amygdala connectivity to regions including the dorsolateral prefrontal cortex (PFC), dorsal anterior cingulate cortex, insula, and cerebellum compared to White participants. No differences were observed in amygdala reactivity to threat. Amygdala connectivity was associated with 3-month PTSD symptoms, but the associations differed by racial/ethnic group and were partly driven by group differences in structural inequities. The present findings suggest variability in tonic neurophysiological arousal in the early aftermath of trauma between racial/ethnic groups, driven by structural inequality, impacts neural processes that mediate susceptibility to later PTSD symptoms.

## Introduction

Responses to traumatic stress vary depending on the level of prior burden individuals bring to the traumatic event. Access to wealth and economic resources, for example, are known protective factors that help to ameliorate long-term social, emotional, and financial burdens of trauma [1, 2]. In the United States, there are clear racial and ethnic inequities in the distributions of certain socioeconomic protective factors including educational attainment, employment, and income [3, 4]. Limited research has focused on how these observable inequities may manifest as race-related differences in traumatic stress responses and may interact with neurobiological mechanisms of trauma and stress-related disorder development. Characterization of potential race-related variation in post-trauma neurophysiology and trauma outcome relationships is important for generating equitable research and clinical approaches for treatment and prevention.

Neurobiological investigations have found consistent evidence that threat neurocircuitry, and particularly the amygdala, plays a significant role in susceptibility to adverse posttraumatic outcomes like posttraumatic stress disorder (PTSD) [5–7]. The amygdala is essential for learned threat responses, and it directly mediates expression of the skin conductance response (SCR) to threat [8, 9]. Both amygdala reactivity and SCR to threat are altered in individuals diagnosed with PTSD [10, 11]. Specifically, amygdala hyperreactivity to threat [6, 12] and heightened expression of SCRs in the early aftermath of trauma [13, 14] are each associated with later PTSD symptom severity. Recent work demonstrates that variability in amygdala and prefrontal cortex (PFC) activity, and functional and structural connectivity, are associated with later PTSD symptoms after trauma which may reflect reduced top-down regulation of amygdala reactivity [12, 15–19]. The present literature, therefore, suggests that amygdala function and related psychophysiological responses are potential neurobiological markers of trauma-related psychopathology.

Despite the potential for an amygdala-based neural marker of PTSD susceptibility, very limited work to date has investigated potential race/ethnicity-related variability—and the role of social inequities—in these findings. Minoritized groups are more likely to have previous exposure to adverse events throughout development which are known to affect amygdala function [20]. Prior research has demonstrated lower SCRs and startle responses in Black individuals with both typical and PTSD samples [21–23]. However, the prior work gave limited consideration to the potential effects of structural inequities which may contributed to race-related differences in physiological responses. Recent evidence suggests that disparate exposures to negative life experiences throughout development drives both lower amygdala reactivity and SCRs to threat in Black individuals compared to white individuals [24]. Further, prior work observed that greater neighborhood disadvantage is associated with greater connectivity of the amygdala and inferior parietal lobule [25]. The extant literature thus suggests that racially/ethnically minoritized individuals may show adaptive counter-regulatory amygdala dynamics (e.g., emotional blunting) to compensate for greater life stress. The race-related structural inequities may partially contribute to recently observed race-related differences in posttraumatic symptoms in the early aftermath of trauma [26, 27]. However, to the best of our knowledge, no prior work has directly investigated racial/ethnic differences in connectivity of threat neurocircuitry in the early aftermath of trauma and the potential contributions of structural inequities.

The present multi-site study investigated potential racial/ethnic differences in neurophysiological reactivity and connectivity that may be related to posttraumatic dysfunction through an exploratory secondary analysis of the AURORA study. We assessed peripheral expression of the emotional response to threat via skin conductance and startle responses during acquisition of conditioned threat. We also investigated amygdala reactivity to social threat (passive viewing of fearful and neutral faces) and connectivity during rest. We hypothesized that racial/ethnic differences would be observed in physiological arousal and amygdala reactivity during threat such that participants from racially/ethnically-minoritized groups would show lowered threat reactivity compared to white participants. We further anticipated differences between racial/ethnic groups in amygdala connectivity patterns. In addition, we suspected that racial/ethnic variability in amygdala connectivity patterns would be associated with later reported posttraumatic dysfunction at 3 and 6-months after the index trauma. Finally, we assessed if observed race-related neurophysiological differences were accounted for by racial inequities in socioeconomic factors (e.g., area deprivation or income). The findings of the present study highlight important race-related variability in brain circuits related to PTSD development and have significant implications for the usage of neural targets for prediction and treatment of trauma and stress-related disorders.

## Methods and materials

Data for the present analyses were obtained as part of the AURORA Study, a multisite longitudinal study of adverse neuropsychiatric sequelae. Details of the larger AURORA project are described elsewhere [28]. Briefly, trauma-exposed participants were recruited from 22 Emergency Departments (EDs) from across the United States. Trauma was defined as a medical incident requiring admission to the ED, and participants who experienced events such as a motor vehicle collision, high fall (>10 feet), physical assault, sexual assault, or mass casualty incidents were included in the study. Other trauma exposures were also qualifying if: (a) the individual responded to a screener question that they experienced the exposure as involving actual or threatened serious injury, sexual violence, or death, either by direct exposure, witnessing, or learning about the trauma and (b) the research assistant agreed that the exposure was a plausible qualifying event. Trauma was a necessary inclusion criterion for the present study, and no participants without trauma were included. Data were collected for 436 participants recruited between 09/25/2017 and 07/31/2020 who had an MRI and physiological data collection approximately 2-weeks after trauma exposure. A subset of participants in the current report were also included in earlier MRI analyses from the AURORA study though the analyses here are distinct [7, 29, 30]. The present analyses were focused on racial/ethnic differences in early amygdala reactivity/connectivity, and we thus excluded participants who did not have corresponding fMRI data (*n* = 55). Participants were also excluded listwise on the basis of motion or technical issues during task-fMRI (*n* = 74) or rs-fMRI (*n* = 59) (see below and supplement) leaving *n* = 295 participants with complete MRI data of acceptable quality. Participants self-reported their race/ethnicity and were coded into four categories of “Hispanic (“Hispanic”; *n* = 50)”, “non-Hispanic White (“White”; *n* = 98)”, “non-Hispanic Black (“Black”; *n* = 135)”, “non-Hispanic other-race (“Other”; *n* = 11)”, and one participant with no reported race/ethnicity. For the present analyses, we also excluded participants from the “other” or unreported racial category due to small sample size that may impact statistical analyses. In total, 283 participants were included in the analyses (Table 1). All participants gave written informed consent as approved by each study site’s Institutional Review Board.

**Table 1:** Demographic characteristics of the sample.

	Total	Hispanic	White	Black
	M (SD) or N (%)	M (SD) or N (%)	M (SD) or N (%)	M (SD) or N (%)
Age	33.87 (12.47)	32.38 (12.03)	35.87 (13.65)	32.98 (11.62)
Sex at birth				
Male	103 (36.40%)	25 (50%)	34 (34.69%)	44 (32.59%)
Female	180 (63.60%)	25 (50%)	64 (65.31%)	91 (67.41%)
Marital History				
Current/Previous Marriage	87 (30.74%)	17 (34%)	38 (38.78%)	32 (23.70%)
Never Married	195 (68.90%)	32 (64%)	60 (61.22%)	103 (76.30%)
Missing	1 (0.35%)	1 (2%)	0 (0%)	0 (0%)
Education				
High School or less	96 (33.92%)	23 (46%)	22 (22.45%)	51 (37.78%)
Some college or more	187 (66.08%)	27 (54%)	76 (77.55%)	84 (62.22%)
Missing	0 (0%)	0 (0%)	0 (0%)	0 (0%)
Currently Employed				
No	74 (26.15%)	14 (28%)	27 (27.55%)	33 (24.44%)
Yes	173 (61.13%)	27 (54%)	61 (62.24%)	85 (62.96%)
Missing	36 (12.72%)	9 (18%)	10 (10.20%)	17 (12.59%)
Income				
<=$35 K Yearly	104 (36.75%)	13 (26%)	46 (46.94%)	45 (33.33%)
>$35 K Yearly	142 (50.18%)	29 (58%)	40 (40.82%)	73 (54.07%)
Missing	37 (13.07%)	8 (16%)	12 (12.24%)	17 (12.59%)
Area Deprivation Index (Nationally Ranked)	57.48 (30.70)	46.64 (25.54)	44.05 (24.02)	71.24 (30.02)

### Demographic and psychometric data collection

Initial participant demographic and psychometric data were collected after admission to the ED which included trauma exposure type, participant marital status, income, education level, and employment. Participants’ home address was geocoded to derive an area deprivation index (ADI) to reflect neighborhood disadvantage [31]. Participants’ posttraumatic symptoms were assessed within the ED (i.e., a retrospective report in the past 30 days prior to trauma), 2-weeks, 8-weeks, 3-months, and 6-months after trauma exposure. In the present analyses, we focused on potential associations of 2-week fMRI measures with 3- and 6-month symptoms. The 3- and 6-month assessments queried participant symptoms in the past 30 days. PTSD symptoms were assessed using the PTSD Checklist for DSM-5 (PCL-5) [32], a 20-item self-report questionnaire on trauma symptom expression and severity. Depression symptoms were assessed using the Patient-Reported Outcomes Measurement Information System (PROMIS) Depression instrument from the PROMIS short form 8b [33]. T-scores were derived from total responses to eight items scored on a Likert scale from 1 (never) to 5 (always). Anxiety symptoms were assessed using four items from the PROMIS Anxiety Bank [33]. Prior life trauma was assessed using the Life Events Checklist version 5 [34]. Participant’s prior trauma exposure was defined by (a) happened directly, (b) witnessed, (c) happened to someone close to them, or (d) exposed to details due to their occupation. Responses to all questions were summed to derive a prior trauma index.

### Psychophysiological responses to threat

Psychophysiological data were collected during a Pavlovian fear conditioning procedure within a day of the MRI session and collected outside of the MRI scanner described in prior reports [35–37]. Briefly, a shape on a computer screen (a blue square; CS+) was repeatedly paired with an aversive unconditioned stimulus (US) (140 psi airblast to the larynx, 250 ms duration). A different shape (a purple triangle; CS−) was never paired with the aversive stimulus. The paradigm included a 108 dB white noise startle probe that elicited the eyeblink startle response. The startle probe was presented during CS+ and CS− trials, and on its own (noise alone [NA] trials) to assess individual baseline startle response. Following habituation, acquisition consisted of three conditioning blocks with four trials of each type (NA, CS+ paired with US, CS-) in each block, for a total of 12 trials of each type. Ten minutes after acquisition, the extinction phase consisted of four blocks with four trials of each type (CS+, CS−, NA), wherein the airblast never occurred. There were a total of 16 trials of each type during extinction (20 min in duration). Given the focus of the present report on amygdala and threat reactivity, we focused on baseline startle response (EMG activity to the probe during noise alone), tonic skin conductance level (SCL), and fear-potentiated startle (FPS)/SCRs to the CS+ and CS− during the acquisition blocks. For statistical analyses, we excluded EMG and SCL/SCR data if scores were equal to or above 3 standard deviations from the sample mean (individually for each data-type/contrast).

#### Magnetic resonance imaging

Task-fMRI, rs-fMRI, and anatomical MRI data were collected across five sites with relatively harmonized acquisition parameters (Table S1). Results included in this manuscript come from preprocessing performed using FMRIPREP version stable 1.2.2 [1, 2, RRID:SCR_016216], a Nipype [3, 4, RRID:SCR_002502] based tool as in our prior reports [7, 29, 30]. Further processing information is provided in the supplement.

### Task-fMRI of amygdala reactivity

To index neural reactivity to threat, participants completed an emotional reactivity task designed to probe reactivity to social threat cues via passive view of fearful and neutral facial expressions. The task is described in prior work [11, 12, 38]. Briefly, faces from the Ekman faces library were presented in a block design (15 fear blocks and 15 neutral blocks, 10 s rest in between) presented in a pseudorandom order. The order was counterbalanced across participants. SPM12 was used for the initial statistical models after using ICA-AROMA as part of the FMRIPREP pipeline [39, 40]. Emotion blocks were modeled with separate boxcar functions representing the onset and 8 s duration of each block, convolved with a canonical hemodynamic response function. Separate regressors for white matter, cerebrospinal fluid and global signal were included to account for motion/physiological noise. Amygdala reactivity from the 1st level contrasts of fearful – neutral face conditions was extracted from the left and right medial amygdala defined by the Brainnetome atlas [41] and used in statistical analyses (see statistical analysis section).

### Resting-state amygdala connectivity

Following ICA-AROMA, the rs-fMRI data were further processed within the Analysis for Functional NeuroImages (AFNI) program 3dTproject to perform linear detrending, censoring of non-steady state volumes identified by FMRIPREP, bandpass filtering (0.01–0.1 Hz), and regression of white matter, corticospinal fluid, and global signal to account for potential physiological noise. The mean fMRI signal time-course was extracted separately from the left and right medial amygdala defined by the Brainnetome atlas [41] and Z-transformed Pearson correlation coefficients were calculated between each ROI and the rest of the brain (i.e., two voxel-wise connectivity maps for left/right amygdala per participant). Group-level statistical modeling was completed in AFNI using the separate voxelwise connectivity maps.

#### Statistical analyses

Statistical analyses were completed using IBM SPSS version 24, the JASP Statistical Package (https://jasp-stats.org/), and the Analysis of Functional NeuroImages (AFNI) software package [42]. Demographic data such as grade-level, employment, marital status, and income were dummy-coded as per our prior analyses [26]. Univariate ANOVAs assessed racial/ethnic differences in tonic SCLs and baseline startle responses. Post-hoc pairwise comparisons were completed for significant omnibus effects with adjustments to degrees of freedom for inequality of variance between groups were completed when a significant violation was detected (e.g., Levene’s test). Repeated-measures ANOVAs assessed racial/ethnic differences in amygdala activity for the fearful–neutral contrast. For ANOVA models, given the pronounced differences in prior trauma exposure (see results), we completed sensitivity analyses with prior trauma exposure as a covariate. For non-significant planned post-hoc comparisons, we ran confirmatory equivalence tests (described in the supplement). Voxelwise group-level models were completed using 3dMVM [43] in AFNI that included a factor for racial/ethnic group. Due to collinearity between race/ethnicity and site/scanner (as well as missing racial/ethnic categories for some sites), we did not include a covariate of site/scanner in any analyses. For completeness, we completed an additional 3dMVM focusing on the effects of scanner to determine if there was overlap in the observed regions for our primary analysis. In addition, quality control metrics of the fMRI data by site and by racial group are provided in the supplement (see supplementary results; Figures S1 and S2). A gray matter mask that included subcortical areas and the cerebellum was applied to the data. Cluster-based methods for multiple comparison correction were applied to determine the voxel extent *k* needed at a cluster forming threshold of *p* = 0.005 to maintain α = 0.05. Specifically, 3dFWHMx was applied to the 1^st^-level contrasts of the preprocessed rs-fMRI data to derive the autocorrelation function parameters for 3dClustSim (10,000 iterations). The minimum *k* for analyses of the rs-fMRI data was 99 voxels. Given our strong a priori hypotheses about amygdala reactivity during faces task, we also extracted beta values for left and right medial amygdala from the Brainnetome atlas for statistical analysis in SPSS. We further completed univariate analyses of covariance (ANCOVA) to determine if racial/ethnic variability in amygdala connectivity patterns were related to differential outcomes in PTSD, depression, and anxiety symptoms at 3 or 6-months. ANCOVAs included between subject factors for racial/ethnic group and continuous covariates for posttraumatic assessment (one for each type and timepoint) to assess each effect for each connectivity pattern. We applied Benjamini-Hochberg false discovery rate corrections for each analysis within each posttraumatic assessment (i.e., correcting for 14 tests –7 connectivity patterns x 2 timepoints for PTSD, depression, and anxiety separately). Finally, to estimate the effect of racial/ethnic disparities on brain connectivity after accounting for demographic factors, we completed parallel mediation models to determine if demographic factors (marital status, income, education, employment, prior trauma, and area deprivation) mediated race-related differences in amygdala connectivity patterns.

## Results

### Demographic characteristics

Demographic data by racial/ethnic group are reported in Table 1. We observed significant differences in education level [χ^2^ = 9.90, *p* = 0.007], income [χ^2^ = 7.47, *p* = 0.023], and marital status [χ^2^ = 6.46, *p* = 0.040]. White participants tended to have more education, while Black participants were more often unmarried and—along with Hispanic participants—had lower income. No significant difference was observed in employment within the sample [χ^2^ = 0.59, *p* = 0.745]. A significant difference in the area deprivation index (ADI) was observed between the groups [F(2,280) = 31.73, *p* < 0.001]. Post-hoc comparisons showed no differences between Hispanic and White participants, but significantly greater ADI in Black compared to Hispanic [t(179) = 5.42, *d* = 0.91, *p* < 0.001] and White participants [t(221.58) = 8.06, *d* = 1.07, *p* < 0.001]. We further observed a significant effect of prior trauma [F(2,220) = 3.12, *p* = 0.046]. Post-hoc comparisons revealed White participants had greater prior trauma exposure than Black participants [t(182) = 2.47, *d* = 0.37, *p* = 0.015] consistent with prior research from the AURORA study [26]. Broad-class trauma exposures by group are presented in Table S2. Given the significant group differences in prior trauma exposure, we conducted sensitivity analyses for significant effects of racial/ethnic group.

### Racial/ethnic differences in tonic physiological arousal but not reactivity during threat learning

A paired-samples t-test revealed significantly greater SCRs to the CS + than the CS- [t(134) = 2.67, *d* = 0.23, *p* = 0.009] during acquisition, confirming successful fear conditioning across the full sample. A one-way ANOVA revealed significant racial/ethnic differences in tonic SCLs [F(2,130) = 7.78, *p* < 0.001]. In sensitivity analyses that included a covariate for prior trauma exposure (i.e., ANCOVA), tonic SCL still differed by racial/ethnic group [F(2,109) = 5.90, *p* = 0.003]. Posthoc pairwise comparisons revealed Black participants showed significantly lower tonic SCL compared to White participants [t(55.76) = 3.36, *d* = 0.69, *p* = 0.001] (Figure S3). These effects survive a Bonferroni correction (criterial *p* = 0.05/3 = 0.016). No difference was observed between Hispanic and White, or Hispanic and Black, participants (all *p* > 0.05). Subsequently, a repeated measures ANOVA did not reveal a significant main effect of racial/ethnic group (*p* = 0.216) or racial/ethnic group by stimulus-type interaction (*p* = 0.820) on SCRs during acquisition. The main effect of stimulus type remained significant [F(1,132) = 4.93, *p* = 0.03]. Thus, racial and ethnic groups differed in baseline levels of peripheral arousal, but did not show differences in physiological reactivity during threat acquisition after trauma exposure.

We next investigated if tonic SCL was related to demographic factors (education, employment, marital status, income, prior trauma, and ADI). Tonic SCL and ADI were correlated at trend-level (*r* = −0.16, *p* = 0.076). Tonic SCL was not associated with other demographic variables. Given the difference in tonic SCL was driven by differences between White and Black participants, we focused a follow-up parallel mediation analysis on these groups. A parallel mediation analysis revealed significant total [Z-stat_c_ = −4.01, *p* < 0.001] and direct [Z-stat_c’_ = −3.18, *p* = 0.001] effects of racial group, but there was not a significant indirect effect [Z-stat_ab_ = −0.36, *p* = 0.719]. These data suggest the indexed structural inequities do not directly mediate the differences in tonic skin conductance between Black and White participants.

### Racial/ethnic differences in baseline, but not fear-potentiated, startle during threat learning

A paired-samples t-test revealed significantly greater FPS response to the CS+ than the CS- [t(208) = 7.80, *d* = 0.54, *p* < 0.001] during acquisition. A one-way ANOVA revealed significant racial/ethnic differences in baseline startle responses [F(2,213) = 5.98, *p* = 0.003]. In sensitivity analyses that included a covariate for prior trauma exposure (i.e., ANCOVA), baseline startle responses still differed by racial/ethnic group F(2,166) = 4.05, *p* = 0.019. Post-hoc pairwise comparisons revealed Black participants showed significantly lower baseline startle compared to White participants [t(157.58) = 3.31, *d* = 0.50, *p* = 0.001] (Figure S3). These effects survive a Bonferroni correction (criterial *p* = 0.05/3 = 0.016). No difference was observed between Hispanic and White, or Hispanic and Black, participants (all *p* > 0.05). Subsequently, a repeated measures ANOVA did not reveal a significant main effect of racial/ethnic group (*p* = 0.732) or racial/ethnic group by stimulus-type interaction (*p* = 0.910) on FPS responses during acquisition. The main effect of stimulus type remained significant [F(1,206) = 51.55, *p* < 0.001]. These data further confirm race-related differences in general physiologic arousal, but not differences in threat reactivity.

We next investigated if baseline startle response was related to demographic factors (education, employment, marital status, income, prior trauma, and ADI). Baseline EMG and ADI were significantly correlated (*r* = −0.22, *p* = 0.001). Given the difference in baseline EMG was driven by differences between White and Black participants, we focused a follow-up parallel mediation analysis on these groups. A parallel mediation analysis revealed significant total [Z-stat_c_ = −3.38, *p* = 0.008] and a significant indirect effect [Z-stat_ab_ = −1.98, *p* = 0.048], but not a significant direct [Z-stat_c’_ = −1.75, *p* = 0.080] effects of racial group. These data suggest structural adversity mediates differences in baseline startle responses between Black and White trauma survivors.

### Racial/ethnic groups do not differ in amygdala reactivity to fearful faces

A mixed measures ANOVA revealed no main effect of racial/ethnic group, and no racial/ethnic group by hemisphere interaction, on amygdala reactivity to threat (Fearful - Neutral faces; *p* > 0.05) (Figure S4). Exploratory post-hoc analyses revealed a difference in left amygdala reactivity between Hispanic and White participants (t(146) = 2.35, *d* = 0.41, *p* = 0.020, uncorrected) that did not survive multiple comparison correction. These data suggest racial/ethnic groups do not differ in amygdala reactivity to threat.

### Racial/ethnic groups differ in basal amygdala to salience network connectivity patterns

**Fig. 1:**
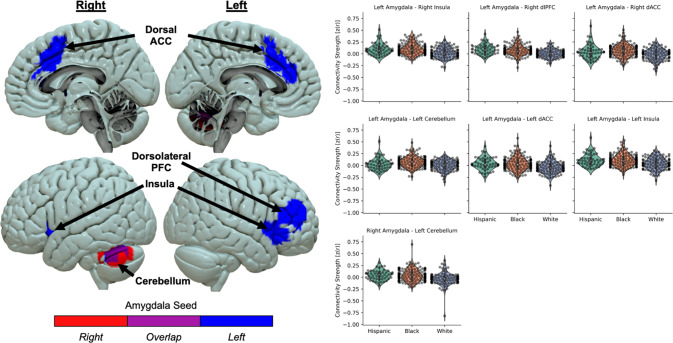
Lower amygdala to salience network connectivity in White, compared to Hispanic and Black, trauma survivors. Several brain regions such as the dorsal anterior cingulate cortex (ACC), dorsolateral prefrontal cortex (PFC), insula, and cerebellum showed racial/ethnic differences in connectivity to both right (red) and left (blue) amygdala. Hispanic (green) and Black (orange) groups showed greater connectivity than White (purple) participants. Violin plots show distribution of participant connectivity strength (dots in overlaid swarm plot) for each group.

General patterns of left and right amygdala connectivity are presented in the supplement (Figure S5). Racial/ethnic-related differences in amygdala connectivity during rs-fMRI are highlighted in Fig. 1. We observed significant differences in connectivity patterns with the left amygdala seed to regions such as cerebellum and dorsolateral PFC, as well as nodes in the canonical salience network, specifically the dorsal anterior cingulate cortex and insula (Table 2). We observed significant differences in connectivity between the right amygdala seed to the cerebellum. Sensitivity analyses including prior trauma exposure as a covariate did not show a significant effect for prior trauma exposure (all *p* > 0.05) and race/ethnicity remained significant (all *p* < 0.05) for the observed clusters. In general, Hispanic and Black participant groups showed higher resting-state connectivity between the amygdala seeds and these nodes than White participants (Table 3). Although left and right amygdala seeds both showed significant race-related connectivity patterns with cerebellum, a subsequent conjunction analysis did not meet a statistically significant cluster extent (*k* = 84). Finally, comparative models focused on effects of scanner did not reveal spatial overlap with the models focused on racial/ethnic group (Figure S6). These results highlight race-related differences in connectivity between the amygdala to major nodes of the salience network that does not appear to be driven by scanner effects.

**Table 2:** Loci of racial/ethnic differences in amygdala connectivity.

Seed	Region	Hemisphere	*k*	F-Statistic	X, Y, Z
Left	Insula	Right	565 (4,520)	6.07	41, 19, −1
		Left	113 (904)	4.29	−37, 14, 4
	Dorsolateral PFC	Right	366 (2,928)	8.66	44, 42, 12
	Dorsal ACC	Right	202 (1,616)	10.44	6, 20, 37
		Left	142 (1,136)	6.59	−9, 29, 27
	Cerebellum	Left	138 (1,104)	8.62	−39, −57, −31
Right	Left Cerebellum	Left	306 (2,448)	8.51	−45, −59, −32

Coordinates are provided in Montreal Neurological Institute (MNI) standard space. F-statistic represents the F-value at the center of mass of the cluster. Cluster size (*k*) expressed as voxels (volume in mm^3^).

**Table 3:** Post-hoc tests of race-related differences in amygdala connectivity.

		Hispanic vs. White		Hispanic vs. Black		White vs. Black	
Amygdala Seed	Node	*t-statistic (Cohen’s d)*	*p-value*	*t-statistic (Cohen’s d)*	*p-value*	*t-statistic (Cohen’s d)*	*p-value*
Left	Right Insula	**3.85 (0.67)**	**<0.001**	−0.13 (−0.02)	0.991	**−5.19 (−0.70)**	**<0.001**
Left	Right DLPFC	**5.07 (0.97)**	**<0.001**	1.33 (0.20)	0.38	**−4.98 (−0.67)**	**<0.001**
Left	Right dACC	2.56 (0.44)	0.029	−1.19 (−0.19)	0.458	**−4.84 (−0.67)**	**<0.001**
Left	Left Cerebellum	1.43 (0.25)	0.329	−2.19 (−0.35)	0.075	**−4.59 (−0.62)**	**<0.001**
Left	Left dACC	3.21 (0.58)	0.004	−0.14 (−0.02)	0.989	**−4.38 (−0.58)**	**<0.001**
Left	Left Insula	**3.40 (0.60)**	**0.002**	0.23 (0.04)	0.97	**−4.16 (−0.55)**	**<0.001**
Right	Left Cerebellum	**3.64 (0.64)**	**<0.001**	0.10 (0.02)	0.994	**−4.63 (−0.59)**	**<0.001**

Bold values indicate post-hoc tests survive a Bonferroni correction (0.05 / 21 comparisons = 0.002) for multiple comparisons.

Next, we completed parallel mediation models to determine if accounting for indices of adversity-mediated race-related differences in amygdala connectivity patterns. Adversity metrics partially mediated the difference in amygdala-to-left insula connectivity between White and Black participants (Table S3). No other indirect effects were significant suggesting these metrics did not mediate race-related differences in amygdala connectivity patterns. Separate correlations and t-tests between the demographic variables and amygdala connectivity patterns are described in the supplement (Table S4).

### Racial/ethnic differences in connectivity and posttraumatic outcomes

**Fig. 2:**
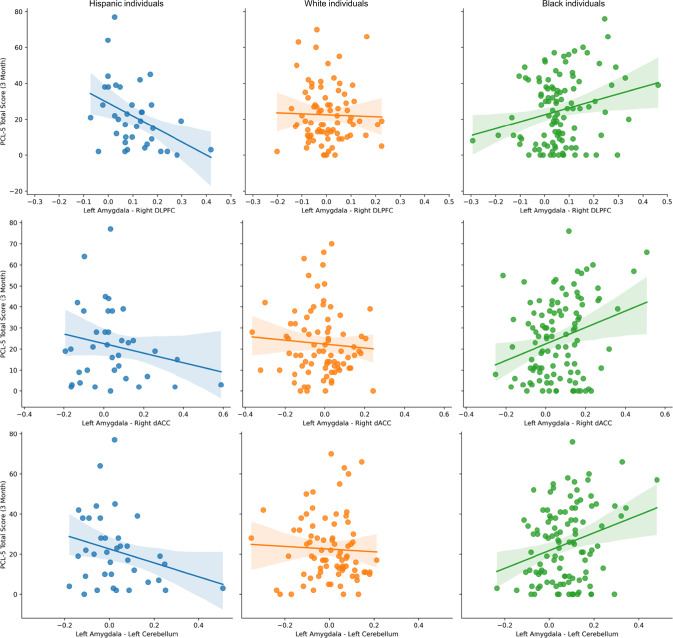
Amygdala connectivity shows differential associations with PTSD symptom development across different races/ethnicities. Racial/ethnic group moderated the relationship between amygdala connectivity to the right dorsolateral prefrontal cortex (DLPFC), right dorsal anterior cingulate cortex (dACC), and left cerebellum. Hispanic individuals (blue) showed negative relationships between connectivity and post-traumatic stress disorder (PTSD) symptoms at 3-months, White individuals (orange) showed an orthogonal relationship, and Black individuals (green) showed a positive relationship. Dots represent individual data points, and the solid lines represent the linear lines of best fit. Shaded areas represent the 95% confidence interval of the linear line of best fit.

Racial/ethnic group by posttraumatic symptom interactions on amygdala connectivity patterns are summarized in Table S5. FDR correction using the Benjamini-Hochberg approach per posttraumatic cluster revealed racial/ethnic group moderated the relationship between connectivity of left amygdala with right DLPFC, right dACC, and left cerebellum, and PCL-5 scores at 3-months (Fig. 2). Specifically, greater connectivity between the amygdala and these regions was associated with lower PCL-5 scores for Hispanic individuals, but greater PCL-5 scores for Black individuals. White individuals showed no relationship between amygdala connectivity and PCL-5 scores. We then re-ran these analyses using residuals from models of demographic factors (i.e., prior trauma, ADI, income, education, marriage, and employment) on amygdala connectivity to determine if accounting for structural inequities affected the relationship. After accounting for structural inequities, only left amygdala to left dACC connectivity was differentially associated with 3-month PCL-5 scores between racial/ethnic groups [F(2,181) = 3.13, *p* = 0.046]. These data suggest neural patterns may predict future PTSD symptom severities differently for racial/ethnic groups and this variability is driven—in part—by structural inequities between groups.

## Discussion

Despite well-documented racial inequities in societal risk and recovery factors for PTSD, limited research has investigated how these inequities may manifest in the neural markers of PTSD susceptibility. The current study used multisite rs-fMRI data from the AURORA study to identify race-related differences in amygdala functional dynamics after trauma and the moderating role of structural inequities. Black and Hispanic individuals displayed heightened connectivity between the amygdala and nodes of the salience network as well as dorsolateral PFC and cerebellum compared to White individuals. Further, Black participants showed lower tonic skin conductance levels (SCLs) and baseline startle responses compared to White participants. There were no racial or ethnic differences in amygdala, skin conductance, or startle reactivity to threat. Accounting for structural inequities attenuated baseline startle responses and the magnitude of racial/ethnic differences in amygdala connectivity. Importantly, these results demonstrate that lower socioeconomic position conveys higher resting amygdala connectivity to the salience network, and that the racial disparities in socioeconomic factors contribute to the appearance of race-related differences in neurophysiological tone. The present findings are critical for the development of generalizable neurobiological markers of susceptibility to trauma and stress-related disorders.

Neurophysiological differences were observed in tonic arousal between racial/ethnic groups, specifically with race-related variability in amygdala connectivity to the insula, dACC, dlPFC, and cerebellum. Of note, the insula and dACC are thought to be part of a salience network that directs attention towards biologically relevant stimuli [44, 45]. Given the role of the amygdala in threat learning and expression, increased amygdala-salience network connectivity may be thought to represent heightened emotional readiness for impending threat that potentiates physiological arousal. Prior research has observed greater amygdala-salience network connectivity in those with PTSD compared to those without, which may suggest that this connectivity pattern is indicative of emotion dysregulation [46]. However, Black participants showed lower SCLs and baseline startle responses compared to White participants indicative of lower tonic physiological arousal. The lower physiological tone is more suggestive of desensitization to threat which is in line with previous neurophysiological research in Black individuals, which has found amygdala sensitization to threat cues as well as lower observed rates of internalizing disorders in Black individuals [47–50]. In fact, prior work has found that increased connectivity between the salience network and other brain regions in those with a history of childhood maltreatment is related to increased psychological resiliency [51]. It is noteworthy however that Hispanic participants were not significantly different from Black or White participants in analyses of SCLs and baseline startle responses. Prior work in non-psychiatric samples has found that Hispanic individuals may show blunted startle responses compared to non-Hispanic individuals [52]. Despite the behavioral differences, Black and Hispanic individuals both showed heightened connectivity of the amygdala compared to White individuals which may suggest groups engage in similar adaptive neural strategies to mitigate the deleterious effects of race/ethnicity-related stressors. The current results may therefore suggest the neurophysiological profiles are indicative of differential emotion regulation approaches wherein Black and Hispanic groups utilize amygdala-salience network connectivity to promote regulated emotion at baseline.

Neuroimaging studies on the brain health consequences of racial discrimination lend some support to the hypothesis of baseline emotion regulation as a correlate of greater amygdala-salience network connectivity in Black individuals. Greater endorsement of discrimination is associated with greater amygdala to dACC and insula (i.e., salience network) connectivity in Black older adults [53]. Further, a prior study found that trauma-exposed Black women with more experiences of racial discrimination had increased response in threat processing network regions that accompanied relatively better performance on an emotional stroop task that included threatening distractors [54]. Relatively less work on the neurobiological consequences of discrimination and race-related stress has been completed in Hispanic individuals although exposed to race/ethnicity-related stressors such as discrimination [55]. Racial discrimination is a component of multi-level racism that is often experienced by minoritized groups. Structural and systemic inequities in income, education, and other socioeconomic factors are considered components of structural racism [56]. One speculative hypothesis then is that the present racial/ethnic differences in neural connectivity are a result of chronic, repeated racism-related stress throughout development. These findings may help to contextualize lower acute and chronic posttraumatic psychopathology symptoms after trauma [26, 57]. Taken together, individuals exposed to factors related to multi-level racism show greater amygdala-salience network connectivity that may allow for greater emotion-regulation during tasks that also contributes to general desensitization, including lower levels of resting peripheral arousal. However, it should also be noted that racial discrimination is associated with increased depressive symptoms [58] and thus more research is needed at the intersection of racism, neurobiology, and psychiatry to fully understand neural associations of racial health disparities. Importantly, the race/ethnicity-related differences in neural connectivity patterns observed herein may also have implications for neuromodulatory-based treatments. For example, dlPFC to amygdala connectivity is a suggested prognostic marker of PTSD, and modulating this connection may be a mechanism for early evidence of transcranial magnetic stimulation (TMS) efficacy in PTSD [7, 17, 59]. However, based on our results, disparate rates of exposure to early life stressors may affect generalizability of such neuromodulatory targets for different racial/ethnic groups particularly when considering additional socioeconomic barriers to treatment.

Of note, we observed no differences in brain or behavioral reactivity to threat after trauma exposure across the racial/ethnic groups. Amygdala reactivity before or in the early aftermath of trauma is predictive of later PTSD symptoms [6, 12] and was a feature of biotypes of posttraumatic sequalae in previous work from the AURORA study [29]. Similarly, SCR and FPS appear to be reproducible physiological markers of PTSD susceptibility [10, 13, 14]. These findings may suggest that measures of threat reactivity obtained relatively soon after trauma may be more generalizable markers of trauma outcomes. However, stimuli used to index amygdala reactivity to threat predominately consisted of white faces which may elicit differing responses from each racial/ethnic group due to in-group/out-group effects [60]. Though similar results were observed using non-racial stimuli during Pavlovian conditioning in the present study, it is possible that a balanced mixed-race stimulus set may have led to different results and may be more ecologically valid. Additional research is needed using balanced stimulus sets to fully explore potential race-related differences in threat reactivity in the early aftermath of trauma.

Several limitations should be noted for the present investigation. First, our sample was limited to racial/ethnic groups of Hispanic, non-Hispanic White, and non-Hispanic Black. The present sampling did not allow for a more nuanced breakdown of racial or ethnic categories which may influence the current findings. Second, the present study only included participants who had experienced a DSM-5 criterion A traumatic event. It is technically and ecologically difficult to recruit previously trauma-unexposed individuals or recruit individuals right before trauma given both the preponderance of trauma in the US and lack of knowledge as to which individuals will soon experience trauma. However, the current and prior work suggests that pre-traumatic psychopathology symptoms vary between racial/ethnic groups, which may be related to pre-traumatic variability in amygdala connectivity. Thus, neuroimaging in the pre-traumatic period, perhaps in pre-post first-responder or military deployment studies, may be useful for understanding race-related differences in neural connectivity and psychiatry disorders. We also note that the socioeconomic factors assessed here may not fully capture the degree of structural inequities between racial/ethnic groups relevant for neuropsychiatric research. Exploratory analyses revealed that ADI—a neighborhood-level measure—was more consistently associated with connectivity patterns than individual socioeconomic measures (Table S3). Emergent research suggests that multidimensional indices of structural inequities, such as those that account for differential exposure to pollutants, may be particularly important for understanding health disparities [61]. Further research is needed that combines granular assessments of structural inequities with neuroimaging in the early aftermath of trauma to understand racial/ethnic disparities in PTSD development. It is also prudent to note that the present analyses were completed as a secondary analysis within the AURORA dataset. Although the largest study of its kind, sampling was limited to five imaging sites which may limit generalizability to participants from other regions. Likewise, the parent study was not specifically designed to investigate other sources of race-related stress (e.g., racial discrimination). Further research including participants from other areas with more in-depth demography is needed to confirm and extend the present findings. Finally, physiological and rs-fMRI data were not collected concurrently in the present study. Continuous psychophysiological measurement during fMRI may allow for better identification of race-related brain-behavior differences important for understanding posttraumatic psychopathology.

In conclusion, the present study identified racial/ethnic variation in amygdala connectivity at rest and tonic physiological arousal during a threat conditioning task, however, no differences were observed between racial/ethnic groups in reactivity to threat. The racial/ethnic variability in amygdala connectivity was also related to expression of PTSD symptoms at 3-months and was partially attributable to the differences in the assessed socioeconomic factors. Our findings have important implications for the development of generalizable neuroimaging markers of posttraumatic dysfunction, and for the usage of neuromodulatory treatments in the aftermath of trauma. Full consideration of the ways in which systemic inequities may produce racial/ethnic variability in neural connectivity after traumatic stress will be necessary for equitable neuroscience-based treatment outcomes.

### Supplementary information

Supplement
