# Supplementary material for: Structural inequities contribute to racial/ethnic differences in neurophysiological tone, but not threat reactivity, after trauma exposure
Source: Mol Psychiatry. 2023 Feb 1;28(7):2975–84. doi: 10.1038/s41380-023-01971-x (PMC10615735; doi:10.1038/s41380-023-01971-x)
Supplement: Supplementary file 1 — Supplement [file 41380_2023_1971_MOESM1_ESM.docx]

**Supplementary Information**

**Supplementary Methods**

*Magnetic resonance image processing*

Results included in this manuscript come from preprocessing performed using FMRIPREP version stable 1.2.2 [1, 2, RRID:SCR_016216], a Nipype [3, 4, RRID:SCR_002502] based tool. Each T1w (T1-weighted) volume was corrected for INU (intensity non-uniformity) using N4BiasFieldCorrection v2.1.0 [5] and skull-stripped using antsBrainExtraction.sh v2.1.0 (using the OASIS template). Brain surfaces were reconstructed using recon-all from FreeSurfer v6.0.1 [6, RRID:SCR_001847], and the brain mask estimated previously was refined with a custom variation of the method to reconcile ANTs-derived and FreeSurfer-derived segmentations of the cortical gray-matter of Mindboggle [21, RRID:SCR_002438]. Spatial normalization to the ICBM 152 Nonlinear Asymmetrical template version 2009c [7, RRID:SCR_008796] was performed through nonlinear registration with the antsRegistration tool of ANTs v2.1.0 [8, RRID:SCR_004757], using brain-extracted versions of both T1w volume and template. Brain tissue segmentation of cerebrospinal fluid (CSF), white-matter (WM) and gray-matter (GM) was performed on the brain-extracted T1w using fast [17] (FSL v5.0.9, RRID:SCR_002823). Functional data were slice time corrected using 3dTshift from AFNI v16.2.07 [11, RRID:SCR_005927] and motion corrected using mcflirt (FSL v5.0.9 [9]). This was followed by co-registration to the corresponding T1w using boundary-based registration [16] with six degrees of freedom, using bbregister (FreeSurfer v6.0.1). Motion correcting transformations, BOLD-to-T1w transformation and T1w-to-template (MNI) warp were concatenated and applied in a single step using antsApplyTransforms (ANTs v2.1.0) using Lanczos interpolation. Frame-wise displacement [19] was calculated for each functional run using the implementation of Nipype. ICA-based Automatic Removal Of Motion Artifacts (AROMA) was used to generate aggressive noise regressors as well as to create a variant of data that is non-aggressively denoised [20]. Many internal operations of FMRIPREP use Nilearn [22, RRID:SCR_001362], principally within the BOLD-processing workflow. For more details of the pipeline see <https://fmriprep.readthedocs.io/en/stable/workflows.html>.

*Confirmatory equivalence independent samples equivalence tests*

Non-significant independent samples t-test were followed up with exploratory equivalence tests. Equivalence tests were completed using the JASP statistical package’s frequentist approach for equivalent independent samples t-tests based on the TOSTER-R package [23]. Student’s or Welch’s test statistic was used depending on whether groups showed inequality of variance (described in the main text). Equivalence regions were set between a Cohen’s *d* of -0.05 to 0.05 at a = 0.05. Results are presented in Table S6. Thus, we cannot reject non-equivalence in the sample, nor can we reject a null hypothesis of group equivalence in threat reactivity.

**Supplemental Results**

*Race/ethnicity and site differences in MRI data quality metrics*

*Faces task*

Differences in quality control metrics by scanner site and by race/ethnicity were analyzed separately for the faces task and the resting state paradigm with Analyses of Variance (ANOVAs). AQI for the faces task differed significantly by scanning site, *F*(4, 278) = 48.75, *p* = 4.83x10^–31^. Pairwise comparisons with Bonferroni adjustments revealed significant differences between the Site 1 and Site 2 (*p* = 0.02), Site 3 (*p* = 5.80x10^–12^), and Site 5 (*p* = 7.59x10^–8^), but not Site 4. Site 2 differed significantly from Site 3 (*p* = 2.07x10^–23^) and Site 5 (*p* = 2.08x10^–7^) but not Site 4. Site 3 differed significantly from Site 4 (*p* = 1.86x10^–22^), and Site 4 differed significantly from Site 5 (*p* = 7.06x10^–8^). There were no statistically significant differences in AQI by race/ethnicity, *F*(2, 280) = 2.32, *p* = 0.10.

There were also significant differences in TSNR between sites, *F*(4, 278) = 47.69, *p* = 1.67x10^–30^. Site 1 differed significantly from Site 3 (*p* = 1.01x10^–9^) and Site 5 (*p* = 1.25x10^–4^). Site 2 also differed significantly from Site 3 (*p* = 1.15x10^–23^) and Site 5 (*p* = 9.98x10^–5^). Site 3 differed significantly from Site 4 (*p* = 4.21x10^–25^) but not Site 5, and Site 4 differed significantly from Site 5 (*p* = 3.43x10^–6^). There was no effect on race/ethnicity on TSNR, *F*(2, 280) = 1.42, *p* = 0.24.

DVARS for the faces task differed significantly by site, *F*(4, 278) = 13.61, *p* = 3.80x10^–10^. Site 1 differed significantly only from Site 3 (*p* = 3.28x10^–3^). Site 2 also differed significantly from Site 3 (*p* = 1.82x10^–9^), and Site 3 differed significantly from Site 4 (*p* = 7.92x10^–8^). There were no significant differences in DVARS by race/ethnicity, *F*(2, 280) = 0.31, *p* = 0.73.

Framewise displacement scores did not differ significantly by site, *F*(4, 278) = 0.65, *p* = 0.63, nor by race/ethnicity, *F*(2, 280) = 0.86, *p* = 0.42.

*Resting state*

In the resting state paradigm, AQI differed significantly by site, *F*(4, 278) = 49.91, *p* = 1.25x10^–31^. Pairwise comparisons revealed that Site 1 differed significantly from Site 3 (*p* = 3.10x10^–3^) and Site 5 (*p* = 0.01; *p*-values Bonferroni adjusted). Site 2 also differed significantly from Site 3 (*p* = 1.88x10^–21^) and Site 5 (*p* = 3.73x10^–10^). Site 3 site differed significantly from Site 4 (*p* = 4.71x10^–26^), and Site 4 differed significantly from Site 5 (*p* = 1.46x10^–13^). There were no statistically significant differences in AQI for resting state by race/ethnicity, *F*(2, 280) = 1.12, *p* = 0.33.

There were significant differences in TSNR by site, *F*(4, 278) = 49.82, *p* = 1.40x10^–31^. Site 1 significantly differed from Site 3 (*p* = 5.60x10^–4^) and Site 5 (*p* = 2.82x10^–3^). Site 2 differed significantly from Site 3 (*p* = 4.06x10^–20^), Site 4, (*p* = 0.02) and Site 5 (*p* = 2.19x10^–9^). Site 3 differed significantly from Site 4 (*p* = 6.82x10^–27^), and Site 4 differed significantly from Site 5 (*p* = 6.30x10^–14^). There were no statistically significant differences in TSNR for resting state by race/ethnicity, *F*(2, 280) = 0.83, *p* = 0.44.

DVARS varied significantly between scanning sites, *F*(4, 278) = 13.03, *p* = 9.71x10^–10^. Site 2 differed significantly from Site 3 (*p* = 6.08x10^–9^) and Site 4 (*p* = 0.01). Site 3 significantly differed from Site 4 (*p* = 4.31x10^–7^) and Site 5 (*p* = 0.03). There were no statistically significant differences in DVARS for resting state by race/ethnicity, *F*(2, 280) = 0.49, *p* = 0.61.

Finally, there were no significant differences in framewise displacement by site (*F*(4, 278) = 1.88, *p* = 0.12) or race/ethnicity(*F*(2, 280) = 0.87, *p* = 0.42).

Table S1. MRI Acquisition parameters by site.

|  | Site1  Siemens TIM 3T Trio  (12 Channel Head Coil) | Site2  Siemens TIM 3T Trio  (12 Channel Head Coil) | Site3  Siemens MAGNETOM 3T Prisma  (20 Channel Head Coil) | Site4  Siemens 3T Verio  (12 Channel Head Coil) | Site5  Siemens MAGNETOM 3T Prisma  (20 Channel Head Coil) |
| --- | --- | --- | --- | --- | --- |
| Modality |  |  |  |  |  |
| T1-weighted | **TR** = 2530ms, **TEs** = 1.74/3.6/5.46/7.32ms, **TI** = 1260ms, **flip angle** = 7, **FOV** = 256mm, **slices** = 176, **Voxel size** = 1mm x 1mm x 1mm | **TR** = 2530ms, **TEs** = 1.74/3.6/5.46/7.32ms, **TI** = 1260ms, **flip angle** = 7, **FOV** = 256mm, **slices** = 176, **Voxel size** = 1mm x 1mm x 1mm | **TR** = 2300ms, **TE** = 2.96ms, **TI** = 900ms, **flip angle** = 9, **FOV** = 256mm, **slices** = 176, **Voxel size** = 1.2mm x 1.0mm x 12mm | **TR** = 2530ms, **TEs** = 1.74/3.65/5.51/7.72ms, **TI** = 1260ms, **flip angle** = 7, **FOV** = 256mm, **slices** = 176, **Voxel size** = 1mm x 1mm x 1mm | **TR** = 2300ms, **TE** = 2.98ms, **TI** = 900ms, **flip angle** = 9, **FOV** = 256mm, **slices** = 176, **Voxel size** = 1.2mm x 1.0mm x 12mm |
| Functional MRI | **TR** = 2360ms, **TE** = 30ms, **flip angle** = 70, **FOV** = 212mm, **slices** = 44, **Voxel size** = 3mm x 2.72mm x 2.72mm, 0.5 mm gap | **TR** = 2360ms, **TE** = 30ms, **flip angle** = 70, **FOV** = 212mm, **slices** = 44, **Voxel size** = 3mm x 3mm x 3mm, 0.5 mm gap | **TR** = 2360ms, **TE** = 29ms, **flip angle** = 70, **FOV** = 212mm, **slices** = 44, **Voxel size** = 3mm x 2.72mm x 2.72mm, 0.5 mm gap | **TR** = 2360ms, **TE** = 30ms, **flip angle** = 70, **FOV** = 212mm, **slices** = 42, **Voxel size** = 3mm x 2.72mm x 2.72mm, 0.5 mm gap | **TR** = 2360ms, **TE** = 29ms, **flip angle** = 90, **FOV** = 210mm, **slices** = 44, **Voxel size** = 3mm x 3mm x 2.5mm, 0.5 mm gap |

Table S2. Broad class trauma types by racial/ethnic group.

| Trauma type | Hispanic | White | Black |
| --- | --- | --- | --- |
| Motor vehicle collision | 36 | 61 | 103 |
| Physical assault | 6 | 9 | 18 |
| Non-motorized collision | 2 | 5 | 2 |
| Fall <10 feet | 2 | 8 | 4 |
| Animal-related | 2 | 6 | 1 |
| Other* | 2 | 9 | 7 |

*Other category also includes AURORA broad class trauma types of a) Sexual Assault, b) Fall (greater than or equal to 10 feet), c) Burns, and d) Incident causing trauma stress exposure to many people.

Table S3. Parallel mediation analyses of adversity on amygdala connectivity.

|  |  | Total effect | | Indirect Effect | | Direct Effect | |
| --- | --- | --- | --- | --- | --- | --- | --- |
| Amygdala Seed | **Node** | Estimate | p-value | Estimate | p-value | Estimate | p-value |
|  |  | **White vs. Hispanic** | | | | | |
| Left | Right Insula | -0.64 | < .001 | -0.02 | 0.751 | -0.62 | < .001 |
| Left | Right DLPFC | -0.89 | < .001 | -0.05 | 0.393 | -0.83 | < .001 |
| Left | Right dACC | -0.44 | 0.01 | -0.08 | 0.246 | -0.36 | 0.046 |
| Left | Left Cerebellum | -0.25 | 0.15 | 0.02 | 0.81 | -0.26 | 0.149 |
| Left | Left dACC | -0.56 | < .001 | -0.03 | 0.604 | -0.53 | 0.002 |
| Left | Left Insula | -0.58 | < .001 | 0.00 | 0.973 | -0.58 | < .001 |
| Right | Left Cerebellum | -0.62 | < .001 | 0.07 | 0.264 | -0.69 | < .001 |
|  |  | **Hispanic vs. Black** | | | | | |
| Left | Right Insula | 0.02 | 0.902 | 0.03 | 0.717 | -0.01 | 0.957 |
| Left | Right DLPFC | -0.20 | 0.216 | 0.09 | 0.303 | -0.29 | 0.094 |
| Left | Right dACC | 0.19 | 0.248 | 0.04 | 0.635 | 0.15 | 0.396 |
| Left | Left Cerebellum | 0.35 | 0.032 | 0.03 | 0.735 | 0.32 | 0.069 |
| Left | Left dACC | 0.02 | 0.887 | 0.00 | 0.957 | 0.03 | 0.877 |
| Left | Left Insula | -0.04 | 0.816 | 0.10 | 0.253 | -0.13 | 0.453 |
| Right | Left Cerebellum | -0.02 | 0.914 | -0.04 | 0.623 | 0.02 | 0.9 |
|  |  | **Black vs. White** | | | | | |
| Left | Right Insula | 0.66 | < .001 | 0.04 | 0.571 | 0.62 | < .001 |
| Left | Right DLPFC | 0.64 | < .001 | 0.11 | 0.15 | 0.53 | < .001 |
| Left | Right dACC | 0.63 | < .001 | 0.07 | 0.356 | 0.57 | < .001 |
| Left | Left Cerebellum | 0.60 | < .001 | -0.03 | 0.709 | 0.62 | < .001 |
| Left | Left dACC | 0.55 | < .001 | 0.02 | 0.776 | 0.53 | < .001 |
| Left | Left Insula | 0.54 | < .001 | 0.15 | 0.043 | 0.38 | 0.008 |
| Right | Left Cerebellum | 0.57 | < .001 | 0.02 | 0.814 | 0.55 | < .001 |

Table S4. Associations between sociodemographic factors and rs-fMRI clusters.

| Amygdala Seed | Target Region | Marital Status  *t-statistic (p-value)* | Education  *t-statistic (p-value)* | Employment  *t-statistic*  *(p-value)* | Income  *t-statistic (p-value)* | ADI  *Pearson r (p-value)* |
| --- | --- | --- | --- | --- | --- | --- |
| Left | Right Insula | -1.83 (0.069)# | 1.14 (0.254) | -0.15 (0.882) | **-2.84 (0.005)** | **0.15 (0.015)** |
| Left | Right DLPFC | **-3.07 (0.002)#** | **2.10 (0.036)** | 0.28 (0.778) | **-2.97 (0.003)** | **0.15 (0.014)** |
| Left | Right dACC | -1.38 (0.17) | **2.06 (0.041)** | 0.77 (0.441) | **-2.74 (0.007)** | **0.16 (0.007)** |
| Left | Left Cerebellum | 0.34 (0.732) | **1.08 (0.028)** | 1.57 (0.119) | -1.88 (0.062) | **0.12 (0.047)** |
| Left | Left dACC | 0.62 (0.535) | **2.37 (0.018)** | 1.96 (0.051) | -1.79 (0.075) | **0.13 (0.031)** |
| Left | Left Insula | -1.33 (0.186) | 1.59 (0.113) | 44 (0.663) | **-2.38 (0.018)** | **0.17 (0.005)** |
| Right | Left Cerebellum | 0.00 (0.999) | **2.10 (0.037)** | 1.08 (0.283) | -0.84 (0.4) | 0.10 (0.09) |

Note: #Indicates correction applied for significant Levene’s test of inequality of variance. Bold values indicate association was nominally significant at p < 0.05

Table S5. Race-related differences in associations between rs-fMRI and posttraumatic dysfunction.

|  |  | PTSD | | Depression | | Anxiety | |
| --- | --- | --- | --- | --- | --- | --- | --- |
|  |  | 3-Month | 6-Month | 3-Month | 6-Month | 3-Month | 6-Month |
| Seed | Target | F-Statistic  (FDR corrected p-value) | | F-Statistic  (FDR corrected p-value) | | F-Statistic  (FDR corrected p-value) | |
| Left | Right Insula | 4.07 (0.063) | 1.25  (0.506) | 3.41 (0.123) | 1.25  (0.403) | 3.53  (0.109) | 0.71  (0.629) |
|  | Right DLPFC | **6.59 (0.028)** | 1.20  (0.471) | 4.76 (0.126) | 1.48  (0.403) | 5.44  (0.07) | 0.55  (0.677) |
|  | Right dACC | **4.68 (0.047)** | 0.62  (0.686) | 3.83 (0.107) | 0.711 (0.574) | 3.76  (0.175) | 0.33  (0.772) |
|  | Left Cerebellum | **5.16**  **(0.042)** | 2.12  (0.285) | 4.82 (0.063) | 2.23  (0.22) | 3.76  (0.117) | 1.74  (0.418) |
|  | Left dACC | 0.49 (0.725) | 0.16  (0.855) | 0.97 (0.482) | 0.44  (0.646) | 0.92  (0.622) | 0.10  (0.906) |
|  | Left Insula | 4.10 (0.050) | 0.82  (0.616) | 3.20 (0.100) | 1.42  (0.381) | 3.02  (0.143) | 1.08  (0.597) |
| Right | Left Cerebellum | 0.39 (0.733) | 1.95  (0.29) | 0.68 (0.545) | 3.26  (0.112) | 0.85  (0.601) | 1.42  (0.49) |

Table S6. Equivalence tests for threat reactivity null effects.

|  |  | | **Hispanic vs. White** | |  | |
| --- | --- | --- | --- | --- | --- | --- |
| **Measure** | t-test | p-value | Lower-Bound | p-value | Upper-Bound | p-value |
| Amygdala (r) Reactivity | -1.16 | 0.246 | 0.12 | 0.454 | -2.45 | 0.008 |
| Amygdala (l) Reactivity | -2.35 | 0.02 | -1.05 | 0.852 | -3.64 | < .001 |
| SCR (CS+ - CS-) | -0.1 | 0.924 | 0.69 | 0.248 | -0.88 | 0.192 |
| FPS (CS+ - CS-) | 0 | 0.998 | 0 | 0.5 | -0.01 | 0.5 |
|  |  |  | **Hispanic vs. Black** | |  |  |
| Amygdala (r) Reactivity | -0.66 | 0.507 | 0.6 | 0.276 | -1.92 | 0.028 |
| Amygdala (l) Reactivity | -1.29 | 0.197 | 0.02 | 0.493 | -2.61 | 0.005 |
| SCR (CS+ - CS-) | 0.31 | 0.756 | 1.34 | 0.092 | -0.71 | 0.239 |
| FPS (CS+ - CS-) | 0.29 | 0.775 | 0.29 | 0.386 | 0.28 | 0.611 |
|  |  |  | **White vs. Black** | |  |  |
| Amygdala (r) Reactivity | 0.6 | 0.547 | 2.18 | 0.015 | -0.97 | 0.165 |
| Amygdala (l) Reactivity | 1.39 | 0.166 | 3.08 | 0.001 | -0.29 | 0.385 |
| SCR (CS+ - CS-) | 0.6 | 0.55 | 1.99 | 0.024 | -0.8 | 0.212 |
| FPS (CS+ - CS-) | 0.39 | 0.697 | 0.39 | 0.347 | 0.39 | 0.65 |

Note: Amygdala reactivity was defined as the fearful – neutral contrast. SCR = Skin conductance response. FPS = Fear-potentiated startle. CS = Conditioned stimulus (+ = paired with unconditioned stimulus, - = unpaired with unconditioned stimulus).

Figure S1. Scanner-variability in QC metrics for task-fMRI data.


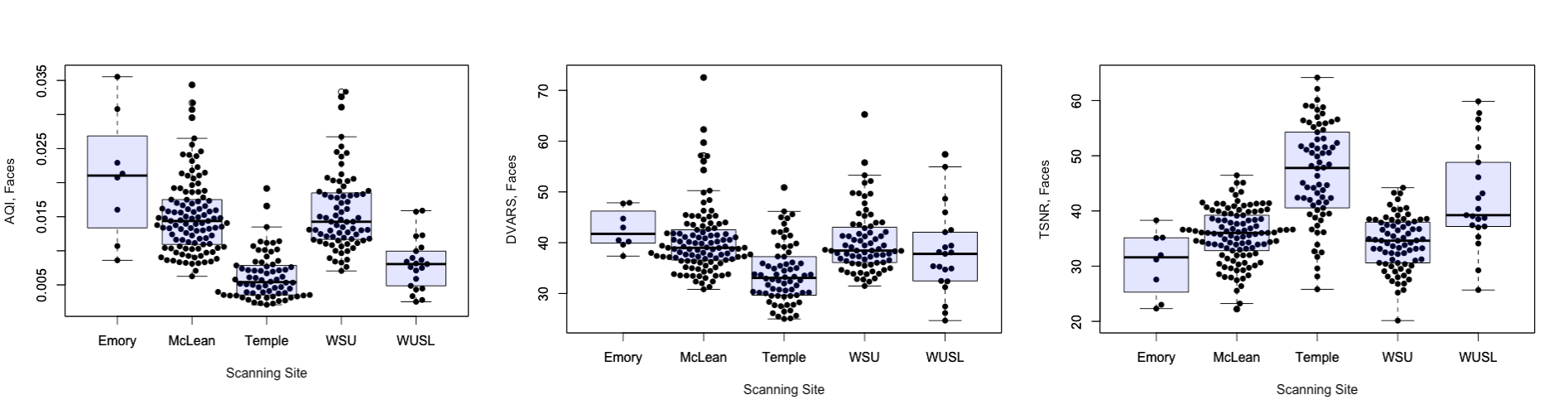


Figure S2. Scanner-variability in QC metrics for rs-fMRI data.


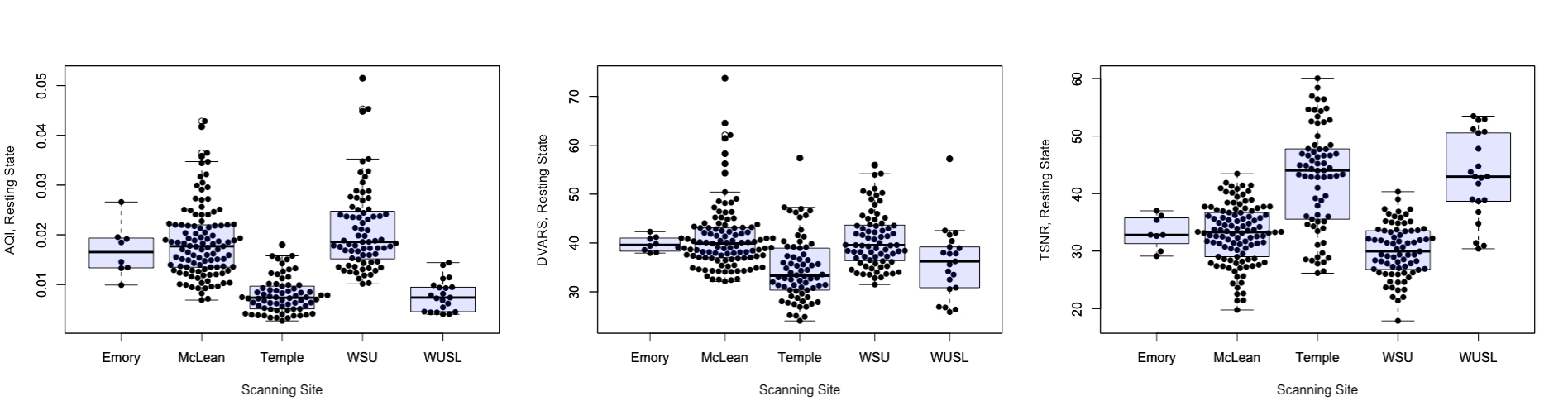


Figure S3. **Differential skin conductance levels and baseline startle in Hispanic, Black and White individuals.** (A) Skin conductance levels were significantly lower in Black compared to White individuals. (B) Baseline startle responses were significantly lower in Black individuals compared to White individuals. Violin plots represent the distribution of data for each group where dots represent individual data points.

**
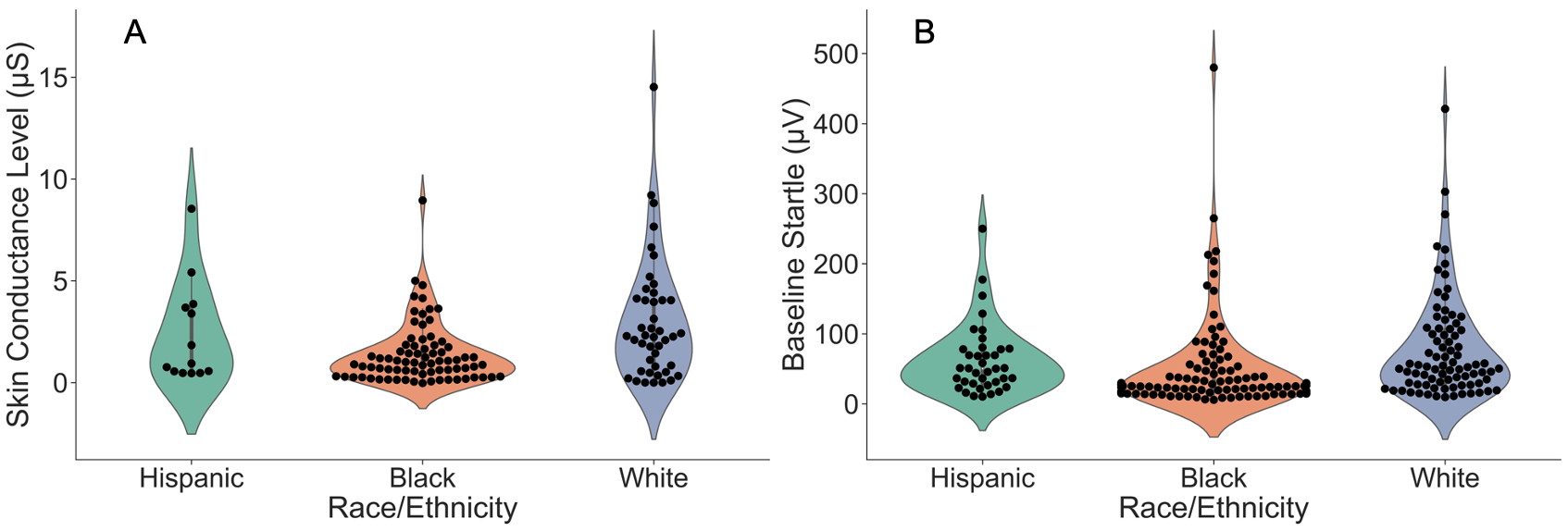
**

Figure S4. **No difference between racial/ethnic groups in threat-related amygdala reactivity.** No significant difference in amygdala reactivity during the passive viewing of fearful and neutral faces was observed between the racial/ethnic groups. Violin plots represent the distribution of data for each group where dots represent individual data points.


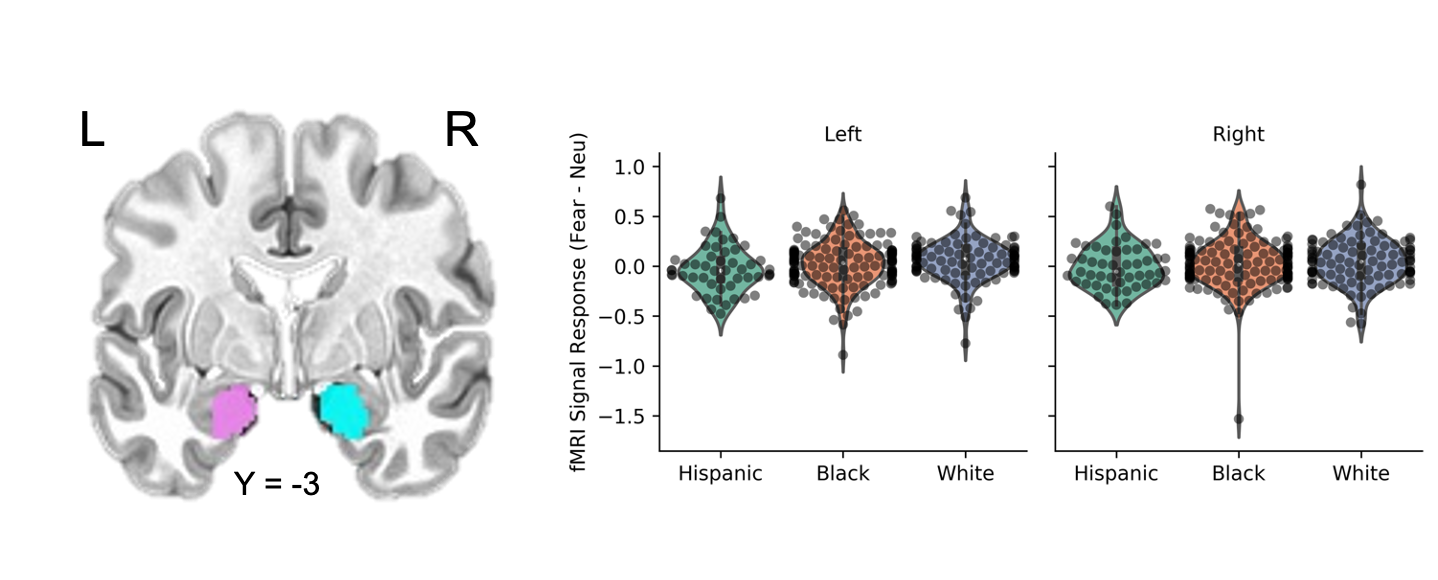


Figure S5. **Whole-brain amygdala connectivity patterns.** Average amygdala connectivity patterns (derived from the intercept of the 3dMVM models) are displayed as F-statistics.


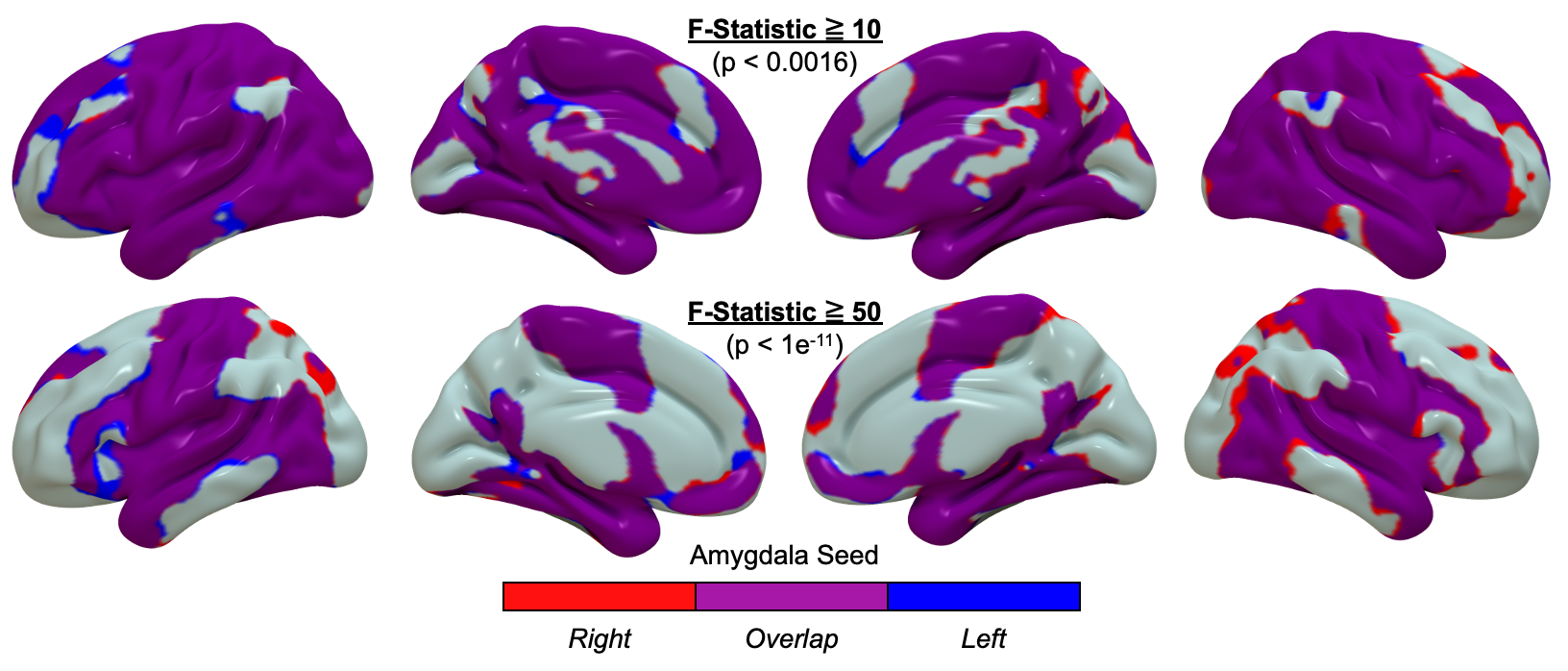


Figure S6. **Comparison of models for scanner effects and race-related effects.** Given the multicollinearity between scan site and participant race/ethnicity, scanner was excluded as a covariate from main models. We completed an additional 3dMVM to model main effects of scanner on left (green) and right (purple) amygdala connectivity. Scan site modulated left amygdala connectivity to the putamen/nucleus accumbens [F-statistic_Peak_ = 6.73, p_corrected_ < 0.05, *k* = 143, (XYZ = -25, 4, -9)] and cerebellum [F-statistic_Peak_ = 6.22, p_corrected_ < 0.05, *k* = 102, (XYZ = 4, -57, -3)]. Scan site also modulated right amygdala connectivity to the right [F-statistic_Peak_ = 6.74, p_corrected_ < 0.05, *k* = 167, (XYZ = 46, -61, -27)] and left [F-statistic_Peak_ = 6.58, p_corrected_ < 0.05, *k* = 138, (XYZ = -21, -73, -19)] cerebellum (a). There was no spatial overlap in models of scanner effects and race-related effects (b).


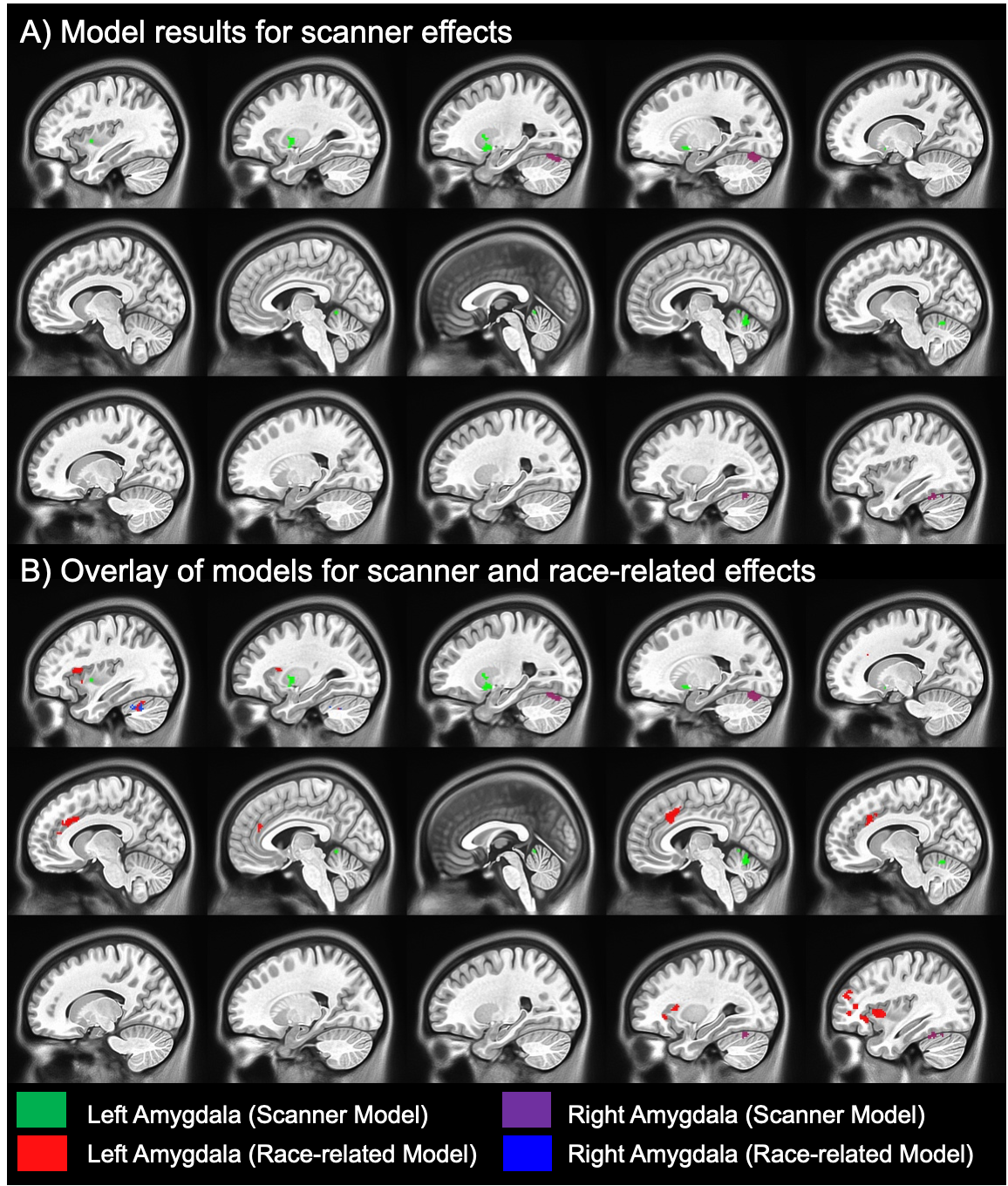


**Supplemental References**

1. Esteban O, Markiewicz CJ, Blair RW, Moodie CA, Isik AI, Erramuzpe A, Kent JD, Goncalves M, DuPre E, Snyder M, Oya H, Ghosh SS, Wright J, Durnez J, Poldrack RA, Gorgolewski KJ. fMRIPrep: a robust preprocessing pipeline for functional MRI. Nat Meth. 2018; doi:[10.1038/s41592-018-0235-4](https://doi.org/10.1038/s41592-018-0235-4)

2. fMRIPrep Available from: [10.5281/zenodo.852659](https://doi.org/10.5281/zenodo.852659).

3. Gorgolewski K, Burns CD, Madison C, Clark D, Halchenko YO, Waskom ML, Ghosh SS. Nipype: a flexible, lightweight and extensible neuroimaging data processing framework in python. Front Neuroinform. 2011 Aug 22;5(August):13. doi:[10.3389/fninf.2011.00013](https://doi.org/10.3389/fninf.2011.00013).

4. Gorgolewski KJ, Esteban O, Ellis DG, Notter MP, Ziegler E, Johnson H, Hamalainen C, Yvernault B, Burns C, Manhães-Savio A, Jarecka D, Markiewicz CJ, Salo T, Clark D, Waskom M, Wong J, Modat M, Dewey BE, Clark MG, Dayan M, Loney F, Madison C, Gramfort A, Keshavan A, Berleant S, Pinsard B, Goncalves M, Clark D, Cipollini B, Varoquaux G, Wassermann D, Rokem A, Halchenko YO, Forbes J, Moloney B, Malone IB, Hanke M, Mordom D, Buchanan C, Pauli WM, Huntenburg JM, Horea C, Schwartz Y, Tungaraza R, Iqbal S, Kleesiek J, Sikka S, Frohlich C, Kent J, Perez-Guevara M, Watanabe A, Welch D, Cumba C, Ginsburg D, Eshaghi A, Kastman E, Bougacha S, Blair R, Acland B, Gillman A, Schaefer A, Nichols BN, Giavasis S, Erickson D, Correa C, Ghayoor A, Küttner R, Haselgrove C, Zhou D, Craddock RC, Haehn D, Lampe L, Millman J, Lai J, Renfro M, Liu S, Stadler J, Glatard T, Kahn AE, Kong X-Z, Triplett W, Park A, McDermottroe C, Hallquist M, Poldrack R, Perkins LN, Noel M, Gerhard S, Salvatore J, Mertz F, Broderick W, Inati S, Hinds O, Brett M, Durnez J, Tambini A, Rothmei S, Andberg SK, Cooper G, Marina A, Mattfeld A, Urchs S, Sharp P, Matsubara K, Geisler D, Cheung B, Floren A, Nickson T, Pannetier N, Weinstein A, Dubois M, Arias J, Tarbert C, Schlamp K, Jordan K, Liem F, Saase V, Harms R, Khanuja R, Podranski K, Flandin G, Papadopoulos Orfanos D, Schwabacher I, McNamee D, Falkiewicz M, Pellman J, Linkersdörfer J, Varada J, Pérez-García F, Davison A, Shachnev D, Ghosh S. Nipype: a flexible, lightweight and extensible neuroimaging data processing framework in Python. 2017. doi:[10.5281/zenodo.581704](https://doi.org/10.5281/zenodo.581704).

5. Tustison NJ, Avants BB, Cook PA, Zheng Y, Egan A, Yushkevich PA, Gee JC. N4ITK: improved N3 bias correction. IEEE Trans Med Imaging. 2010 Jun;29(6):1310–20. doi:[10.1109/TMI.2010.2046908](https://doi.org/10.1109/TMI.2010.2046908).

6. Dale A, Fischl B, Sereno MI. Cortical Surface-Based Analysis: I. Segmentation and Surface Reconstruction. Neuroimage. 1999;9(2):179–94. doi:[10.1006/nimg.1998.0395](https://doi.org/10.1006/nimg.1998.0395).

7. Fonov VS, Evans AC, McKinstry RC, Almli CR, Collins DL. Unbiased nonlinear average age-appropriate brain templates from birth to adulthood. NeuroImage; Amsterdam. 2009 Jul 1;47:S102. doi:[10.1016/S1053-8119(09)70884-5](https://doi.org/10.1016/S1053-8119(09)70884-5).

8. Avants BB, Epstein CL, Grossman M, Gee JC. Symmetric diffeomorphic image registration with cross-correlation: evaluating automated labeling of elderly and neurodegenerative brain. Med Image Anal. 2008 Feb;12(1):26–41. doi:[10.1016/j.media.2007.06.004](https://doi.org/10.1016/j.media.2007.06.004).

9. Jenkinson M, Bannister P, Brady M, Smith S. Improved optimization for the robust and accurate linear registration and motion correction of brain images. Neuroimage. 2002 Oct;17(2):825–41. doi:[10.1006/nimg.2002.1132](https://doi.org/10.1006/nimg.2002.1132).

10. Andersson JLR, Skare S, Ashburner J. How to correct susceptibility distortions in spin-echo echo-planar images: application to diffusion tensor imaging. Neuroimage. 2003 Oct;20(2):870–88. doi:[10.1016/S1053-8119(03)00336-7](https://doi.org/10.1016/S1053-8119(03)00336-7).

11. Cox RW. AFNI: software for analysis and visualization of functional magnetic resonance neuroimages. Comput Biomed Res. 1996 Jun;29(3):162–73. doi:[10.1006/cbmr.1996.0014](https://doi.org/10.1006/cbmr.1996.0014).

12. Jenkinson M. Fast, automated, N-dimensional phase-unwrapping algorithm. Magn Reson Med. 2003 Jan;49(1):193–7. doi:[10.1002/mrm.10354](https://doi.org/10.1002/mrm.10354).

13. Huntenburg JM. Evaluating nonlinear coregistration of BOLD EPI and T1w images. Freie Universität Berlin; 2014. Available from: <http://hdl.handle.net/11858/00-001M-0000-002B-1CB5-A>.

14. Wang S, Peterson DJ, Gatenby JC, Li W, Grabowski TJ, Madhyastha TM. Evaluation of Field Map and Nonlinear Registration Methods for Correction of Susceptibility Artifacts in Diffusion MRI. Front Neuroinform. 2017 [cited 2017 Feb 21];11. doi:[10.3389/fninf.2017.00017](https://doi.org/10.3389/fninf.2017.00017).

15. Treiber JM, White NS, Steed TC, Bartsch H, Holland D, Farid N, McDonald CR, Carter BS, Dale AM, Chen CC. Characterization and Correction of Geometric Distortions in 814 Diffusion Weighted Images. PLoS One. 2016 Mar 30;11(3):e0152472. doi:[10.1371/journal.pone.0152472](https://doi.org/10.1371/journal.pone.0152472).

16. Greve DN, Fischl B. Accurate and robust brain image alignment using boundary-based registration. Neuroimage. 2009 Oct;48(1):63–72. doi:[10.1016/j.neuroimage.2009.06.060](https://doi.org/10.1016/j.neuroimage.2009.06.060).

17. Zhang Y, Brady M, Smith S. Segmentation of brain MR images through a hidden Markov random field model and the expectation-maximization algorithm. IEEE Trans Med Imaging. 2001 Jan;20(1):45–57. doi:[10.1109/42.906424](https://doi.org/10.1109/42.906424).

18. Behzadi Y, Restom K, Liau J, Liu TT. A component based noise correction method (CompCor) for BOLD and perfusion based fMRI. Neuroimage. 2007 Aug 1;37(1):90–101. doi:[10.1016/j.neuroimage.2007.04.042](https://doi.org/10.1016/j.neuroimage.2007.04.042).

19. Power JD, Mitra A, Laumann TO, Snyder AZ, Schlaggar BL, Petersen SE. Methods to detect, characterize, and remove motion artifact in resting state fMRI. Neuroimage. 2013 Aug 29;84:320–41. doi:[10.1016/j.neuroimage.2013.08.048](https://doi.org/10.1016/j.neuroimage.2013.08.048).

20. Pruim RHR, Mennes M, van Rooij D, Llera A, Buitelaar JK, Beckmann CF. ICA-AROMA: A robust ICA-based strategy for removing motion artifacts from fMRI data. Neuroimage. 2015 May 15;112:267–77. doi:[10.1016/j.neuroimage.2015.02.064](https://doi.org/10.1016/j.neuroimage.2015.02.064).

21. Klein A, Ghosh SS, Bao FS, Giard J, Häme Y, Stavsky E, et al. Mindboggling morphometry of human brains. PLoS Comput Biol 13(2): e1005350. 2017. doi:[10.1371/journal.pcbi.1005350](https://doi.org/10.1371/journal.pcbi.1005350).

22. Abraham A, Pedregosa F, Eickenberg M, Gervais P, Mueller A, Kossaifi J, Gramfort A, Thirion B, Varoquaux G. Machine learning for neuroimaging with scikit-learn. Front in Neuroinf 8:14. 2014. doi:[10.3389/fninf.2014.00014](https://doi.org/10.3389/fninf.2014.00014).

23. Lakens, D. (2017). Equivalence tests: A practical primer for t tests, correlations, and meta-analyses. Social psychological and personality science, 8(4), 355-362.
